# Long-term effectiveness of eptinezumab in patients with migraine and prior preventive treatment failures: extension of a randomized controlled trial

**DOI:** 10.1186/s10194-023-01688-w

**Published:** 2023-11-20

**Authors:** Messoud Ashina, Stewart J. Tepper, Astrid Gendolla, Bjørn Sperling, Anders Ettrup, Mette Krog Josiassen, Amaal J. Starling

**Affiliations:** 1 Department of Neurology, Danish Headache Center, Copenhagen University Hospital, Rigshospitalet Glostrup Valdemar Hansen Vej 5, Glostrup, DK-2600 Denmark; 2 Department of Clinical Medicine, University of Copenhagen, Copenhagen, Denmark; 3 The New England Institute for Neurology and Headache, Stamford, CT USA; 4 Praxis Gendolla, Essen, Germany; 5 H. Lundbeck A/S, Copenhagen, Denmark; 6 Mayo Clinic Arizona, Scottsdale, AZ USA

**Keywords:** Efficacy, Most bothersome symptom, Quality of life, Work productivity

## Abstract

**Background:**

Eptinezumab demonstrated efficacy in adults with migraine and prior preventive treatment failures in the placebo-controlled phase of the DELIVER clinical trial; its long-term effectiveness in this population has not yet been reported. The objective of this study was to evaluate the long-term effectiveness of eptinezumab in a migraine patient population during the 48-week extension phase of DELIVER.

**Methods:**

DELIVER was conducted June 1, 2020 to September 15, 2022. 865 adults with migraine, with documented evidence of 2–4 prior preventive migraine treatment failures and with completion of the 24-week placebo-controlled period of DELIVER received eptinezumab (100 or 300 mg) during the dose-blinded extension, either continuing their randomized dose or, if originally receiving placebo, were randomized 1:1 to an eptinezumab dose (100 or 300 mg). A mixed model for repeated measures was used to evaluate changes from baseline in the number of monthly migraine days (MMDs).

**Results:**

Of 865 patients entering the extension (eptinezumab 100 mg, *n* = 433; 300 mg, *n* = 432), 782 (90.4%) completed and 11 (1.3%) discontinued due to an adverse event. Eptinezumab was associated with early and sustained reductions in migraine frequency. Mean MMDs at baseline were approximately 14 days across groups. Mean (standard error) change from baseline in MMDs over the final dosing interval (weeks 61–72) was −6.4 (0.50) with placebo/eptinezumab 100 mg, –7.3 (0.49) with placebo/eptinezumab 300 mg, –7.1 (0.39) with eptinezumab 100 mg, and −7.0 (0.39) with eptinezumab 300 mg. During weeks 61–72, 63–70% of patients demonstrated ≥ 50% reduction in MMDs, and 36–45% demonstrated ≥ 75% reduction. Headache severity and acute medication use reductions, and patient-reported improvements in most bothersome symptom, disease status, quality of life, and work productivity, were observed. Adverse events were generally mild, transient, and similar in frequency/type to previous eptinezumab trials.

**Conclusions:**

The long-term effectiveness and safety/tolerability of eptinezumab in patients with migraine and 2–4 prior preventive treatment failures was demonstrated by high completion rates and migraine-preventive benefits sustained for up to 18 months, implying that eptinezumab is a viable long-term treatment option for patients still seeking successful migraine treatments.

**Trial registration:**

ClinicalTrials.gov (Identifier: NCT04418765; URL: https://www.clinicaltrials.gov/ct2/show/NCT04418765); EudraCT (Identifier: 2019-004497-25; URL: https://www.clinicaltrialsregister.eu/ctr-search/search?query=2019-004497-25).

**Graphical Abstract:**

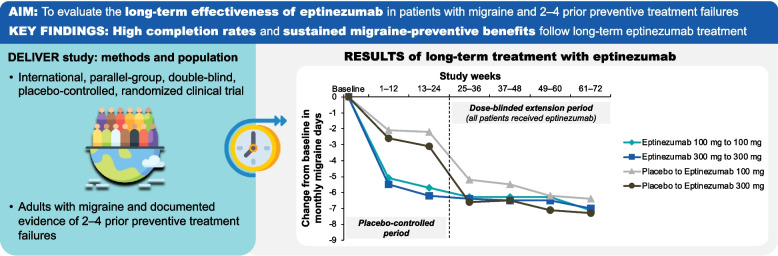

**Supplementary Information:**

The online version contains supplementary material available at 10.1186/s10194-023-01688-w.

## Introduction

Migraine is a chronic neurological disorder that affects an estimated 15% of the global population (over 1 billion people worldwide) [1]. In Europe, the economic consequences of migraine were estimated at €50 billion to €111 billion. Furthermore, direct healthcare costs, which primarily included outpatient care, acute medications and hospitalization, were €1222 per person with migraine in 2011 [2]. Preventive therapy is a crucial aspect of long-term migraine management and could help achieve the goal of reducing utilization of urgent care, outpatient infusions, and inpatient infusions. It is recommended for reducing attack frequency, severity, and duration, as well as for minimizing acute medication use and interictal burden and for enhancing health-related quality of life [3].

Monoclonal antibodies targeting calcitonin gene-related peptide (CGRP) are increasingly used for migraine prevention, particularly in patients who have experienced the failure of older, non-specific oral preventives [4–7]. Eptinezumab is an intravenously (IV)-administered anti-CGRP monoclonal antibody that has demonstrated efficacy in reducing headache frequency and impact across the migraine spectrum, with a fast onset of effect and sustained improvements throughout treatment [8–14]. In the phase 3b DELIVER trial, eptinezumab reduced migraine frequency and health-related impact in adults with 2–4 documented prior preventive treatment failures [4, 15, 16]. The objective of this 48-week, dose-blinded extension of the DELIVER trial was to evaluate the long-term effect of eptinezumab in this difficult-to-treat patient population who were unsuccessfully treated with several classes of older, non-specific oral preventive treatments.

## Methods

### Study design

DELIVER was a multicenter, parallel-group, randomized, double-blind, placebo-controlled phase 3b clinical trial that evaluated the efficacy and safety of eptinezumab for migraine prevention in patients for whom previous preventive treatments failed. The total study duration was 76 weeks, including a screening period of 28‒30 days, a placebo-controlled period of 24 weeks, and a dose-blinded extension period of 48 weeks. The detailed methodology has been published [4], including the protocol and statistical analysis plan; however, key elements are summarized here. The study was conducted in accordance with standards of Good Clinical Practice as defined by the International Conference on Harmonisation and all applicable federal and local regulations. The local review board or a central institutional review board/ethics committee approved all study documentation at each of the 96 study sites. All patients provided written informed consent prior to study participation. Sex and race were self-identified by patients based on fixed categories. DELIVER was registered on ClinicalTrials.gov (NCT04418765) and EudraCT (2019-004497-25).

### Patients

DELIVER enrolled adults (18‒75 years [inclusive]) with migraine and documented evidence of 2–4 previous preventive treatment failures in the prior 10 years. Key inclusion criteria included: onset of migraine (International Classification of Headache Disorders 3rd edition [ICHD-3] [17]) at or before 50 years of age; a history of migraine for ≥ 12 months prior to the screening visit; criteria met for chronic migraine (migraine occurring on ≥ 8 days and headache occurring on > 14 days) or episodic migraine (migraine occurring on ≥ 4 days/month and headache occurring on ≤ 14 days/month) during the screening period; a history of 2–4 previous preventives failing due to inadequate efficacy, safety/tolerability reasons, or contraindications (≥ 1 of the failures had to be due to inadequate efficacy following ≥ 3 months of treatment with the locally recommended dose); and compliance demonstrated with the headache eDiary by data entry for ≥ 24 of the 28 days prior to randomization.

Key exclusion criteria included: failure on a previous treatment targeting the CGRP pathway; confounding and clinically significant pain syndromes; diagnosis of temporomandibular disorder; history or diagnosis of other primary headache types; history of clinically significant cardiovascular disease; uncontrolled and/or untreated psychiatric condition; and use of preventive migraine medication within 1 week prior to the screening visit. Patients with concurrent diagnosis of medication-overuse headache (defined by clinical ICHD-3 criteria [17]) were allowed. Use of acute migraine treatments was allowed provided the dose had been stable for ≥ 12 weeks prior to the screening visit. Medications constituting 2–4 prior preventive treatment failures (beta-blockers: propranolol, metoprolol; anticonvulsants: topiramate, valproate, or divalproex; tricyclic antidepressants: amitriptyline; calcium channel blocker: flunarizine; angiotensin II receptor antagonist: candesartan; medications locally approved for prevention of migraine) were disallowed; however, medications within the same classes as the disallowed preventive migraine medications could be used for other comorbidities.

### Interventions

For the 24-week, placebo-controlled treatment period, patients were randomized to eptinezumab 100 mg, eptinezumab 300 mg, or placebo IV every 12 weeks in a 1:1:1 fashion, with randomization stratified by monthly headache days (MHDs) at baseline (≤ 14 MHDs or > 14 MHDs) and by country. For the 48-week dose-blinded extension, patients who had received eptinezumab in the double-blind phase continued at their randomized dose (100 mg or 300 mg); patients who had received placebo were randomized 1:1 to eptinezumab 100 mg or 300 mg. Codes for randomization in the extension period were assigned at the start of the study. Eptinezumab was administered intravenously once every 3 months for 4 additional doses.

Throughout the study, patients kept a headache eDiary, which comprised evening reports (completed daily regardless of the presence or absence of headaches) and headache reports (completed for each headache that occurred). The latter were used to document headache start and stop times, characteristics, symptoms, severity (mild, moderate, or severe), and acute medication intake in connection with the headache.

### Outcomes

Changes from baseline in the number of monthly migraine days (MMDs) and the percentages of patients achieving ≥ 50% reduction in MMDs (≥ 50% response) and ≥ 75% reduction in MMDs (≥ 75% response) during Weeks 25–36, 37–48, 49–60, and 61–72 were predefined secondary study endpoints. These endpoints were derived from data collected via the eDiary. The change from baseline in the 6-item Headache Impact Test (HIT-6) total score at Weeks 36, 48, 60, and 72 was also a predefined secondary endpoint.

Predefined exploratory endpoints included the change from baseline in the percentage of migraine attacks of severe intensity (Weeks 25–36, 37–48, 49–60, and 61–72); change from baseline in monthly acute migraine medication use; patient-identified most bothersome symptom (PI-MBS) score (Weeks 36, 48, 60, and 72); Patient Global Impression of Change (PGIC) score (Weeks 36, 48, 60, and 72); change from baseline in Migraine-Specific Quality of Life questionnaire (MSQ) subscores (Weeks 36, 48, 60, and 72); change from baseline in EQ-5D-5L visual analog scale (VAS) score (Weeks 36, 48, 60, and 72); and change from baseline in Work Productivity and Impairment, adapted for Migraine (WPAI:M) subscores for absenteeism, presenteeism, work productivity loss, and activity impairment (Weeks 36, 48, 60, and 72). Patient-reported outcomes are described in the Supplemental Methods.

The safety/tolerability of long-term eptinezumab treatment was assessed identically to the primary report and included adverse event monitoring, clinical laboratory testing, physical examinations, electrocardiograms, the Columbia–Suicide Severity Rating Scale, and blood sampling for anti-drug antibody assessment [18].

### Sample size

Sample size calculations were made for the placebo-controlled period and no power calculations were made for the extension period. Based on simulations, an estimated 280 patients per treatment arm (eptinezumab 100 mg, 300 mg, and placebo) provided ≥ 90% power for the primary endpoint and ≥ 68% power for the individual key secondary endpoints [4].

### Statistical analysis

All patients who received ≥ 1 infusion and had a visit in the extension period were included in the safety analyses. All patients who had a valid baseline assessment and a valid assessment of MMDs in the extension period were included in the efficacy analyses. Baseline was the 28-day screening period that occurred before the placebo-controlled treatment period.

A mixed model for repeated measures similar to the one used for the primary endpoint (2-sided; 95% CI) [4] was used to evaluate changes from baseline in the number of MMDs as well as changes from baseline in HIT-6 score. For migraine responder rates, counts and proportions are presented. All exploratory endpoints were analyzed similarly to the secondary endpoints. The eDiary endpoints were derived using prorating if data were captured for ≥ 14 of the 28 days in a 4-week period. Analyses were conducted using SAS software (SAS Institute, Inc., Cary, NC, USA) v9.4 or later.

## Results

### Patients

**Fig. 1:**
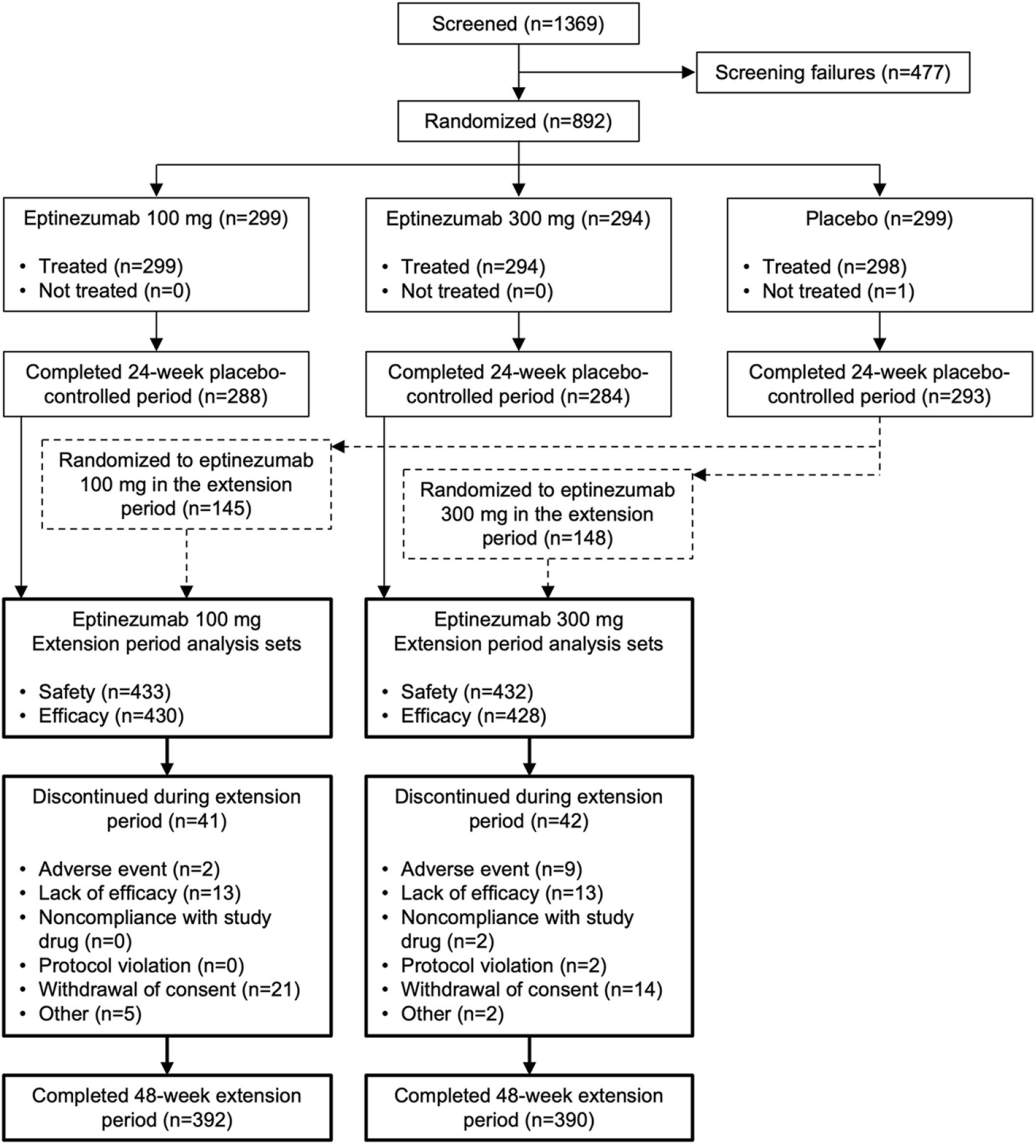
CONSORT diagram of patient disposition.  Safety analyses were conducted in the all-patients-treated set, which included all randomized patients who received ≥ 1 infusion of double-blind study drug and had a visit in the extension period. Efficacy analyses were conducted in the full analysis set, which included all randomized patients who received ≥ 1 infusion of double-blind study drug, had a valid baseline assessment, and had ≥ 1 valid assessment of monthly migraine days in the extension period

Of 892 patients randomized in the 24-week placebo-controlled period, 865 (97.0%) completed that portion of the study, enrolled in this dose-blinded extension period, and comprised the safety analysis set (eptinezumab 100 mg, *n* = 433; eptinezumab 300 mg, *n* = 432). The efficacy analysis set included 858 patients. Of the 865 patients entering the extension period, 782 (90.4%) completed it (Fig. 1). Key baseline characteristics are summarized in Table 1; as the extension population comprised 97% of the entire study population, it seems reasonable to expect that additional parameters not listed here would be similar to those described in the primary report [4].

**Table 1:** Demographic and baseline characteristics of patients enrolled in the dose-blinded extension (efficacy analysis set)

Characteristics	Epti 100 mg / Epti 100 mg (*n* = 286)	Epti 300 mg / Epti 300 mg (*n* = 282)	Placebo / Epti 100 mg (*n* = 144)	Placebo / Epti 300 mg (*n* = 146)
Age, mean (SD), years	44.7 (10.6)	43.0 (10.1)	44.8 (11.0)	43.2 (10.7)
Sex, no. (%)				
Female	264 (92.3)	249 (88.3)	124 (86.1)	132 (90.4)
Male	22 (7.7)	33 (11.7)	20 (13.9)	14 (9.6)
Race, no. (%)				
White	276 (96.5)	270 (95.7)	138 (95.8)	139 (95.2)
Other	0	0	1 (0.7)	1 (0.7)
Unknown	10 (3.5)	12 (4.3)	5 (3.5)	6 (4.1)
MMDs, mean	13.7	13.6	13.7	14.0
Days per month of acute headache medication use, mean	11.1	11.0	11.2	11.2
HIT-6 total score, mean	66.6	66.5	66.4	66.0
MSQ, mean				
Role function restrictive	35.6	35.7	35.6	35.4
Role function preventive	50.3	51.3	51.4	49.8
Emotional function	50.6	48.4	48.4	49.0
EQ-5D-5L VAS, mean	76.0	74.6	75.4	72.6
WPAI, mean				
Absenteeism	11.8	11.7	10.7	14.4
Presenteeism	50.8	53.5	49.7	53.3
Work productivity loss	53.8	57.2	52.8	57.7
Activity impairment	58.3	58.9	58.8	59.0

*Epti *Eptinezumab, *HIT-6 *6-item Headache Impact Test, *MMDs *Monthly migraine days, *MSQ *Migraine-Specific Quality of Life questionnaire, *SD *Standard deviation, *VAS *Visual analog scale, *WPAI:M *Work Productivity Activity Index: Migraine

There was a high level of compliance with the eDiary. The mean rate of missing eDiary data was ~ 10% (mean [SE]: 10.1% [0.65], 100 mg; 9.0% [0.56], 300 mg) during Weeks 25–28, and only increased to ~ 18% during Weeks 69–72 (17.5% [1.04], 100 mg; 18.3% [0.99], 300 mg). The proportion of patients with ≥ 14 days of compliance was 98.5% during Weeks 25–28 and gradually declined to 92.9% at Weeks 69–72.

### Migraine frequency

**Fig. 2:**
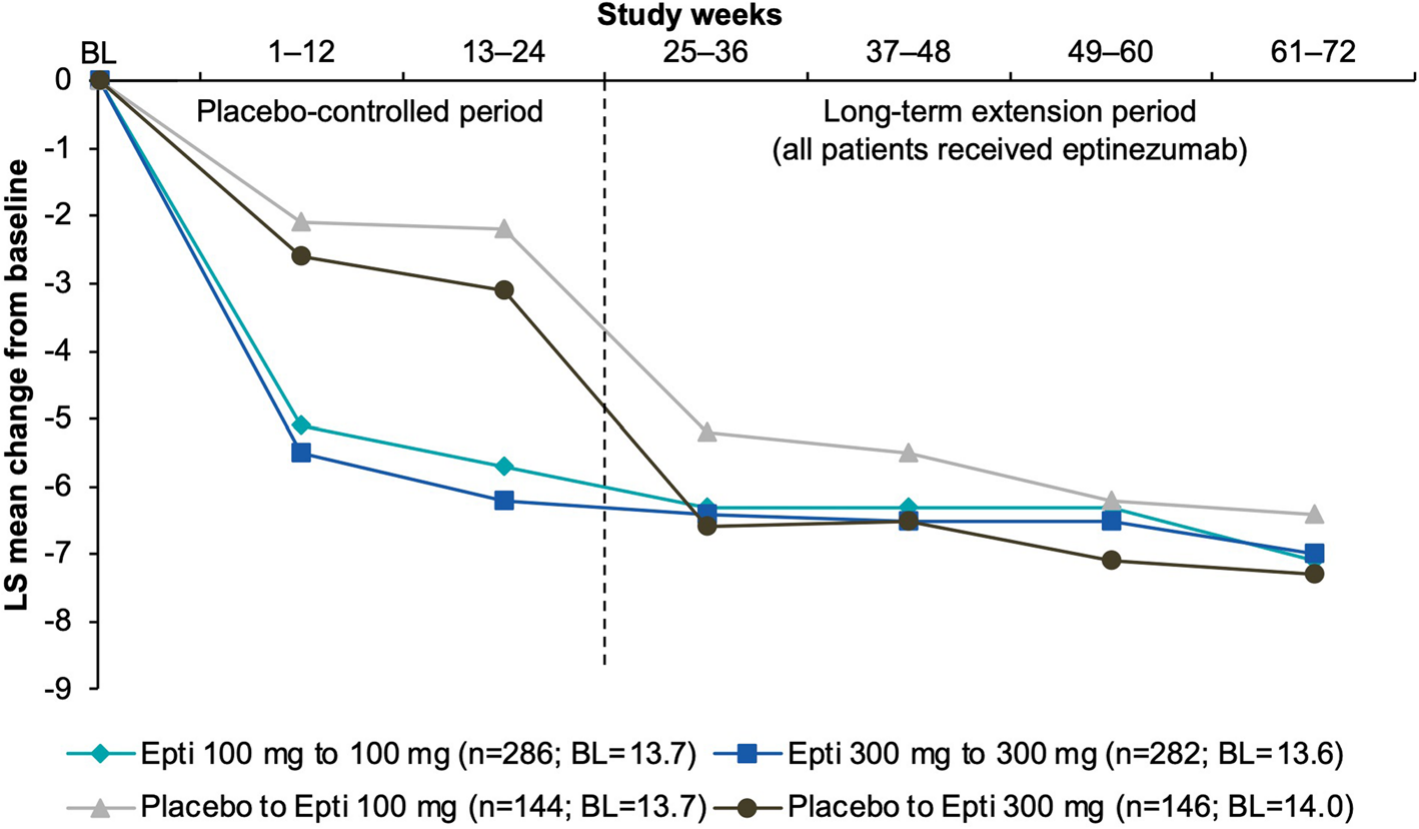
Change from baseline in monthly migraine days (MMRM; efficacy analysis set).  BL, baseline; Epti, eptinezumab; LS, least squares; MMRM, mixed model for repeated measures

Migraine frequency was similar across treatment groups at baseline (~ 14 MMDs). Over the final 4 weeks of the double-blind treatment period (Weeks 21–24), mean (SE) reductions in MMDs were greater in patients that received eptinezumab (−5.6 [0.39] MMDs, 100 mg; −5.7 [0.39] MMDs, 300 mg) than in patients that received placebo (−2.3 [0.50] MMDs, placebo/100 mg and −2.8 [0.50] MMDs, placebo/300 mg) (Supplemental Table 1). For those patients randomized to placebo during the placebo-controlled period, that were only starting treatment with eptinezumab in the long-term extension, the first dose of eptinezumab on average resulted in a steep decrease in MMDs relative to baseline (Weeks 25–28: −5.8 [0.50] MMDs, placebo/100 mg; −7.2 [0.50] MMDs, placebo/300 mg), similar to what was observed with the first eptinezumab treatment in the placebo-controlled period. In all groups, mean reductions in MMDs were sustained through the final assessment (Fig. 2).

**Fig. 3:**
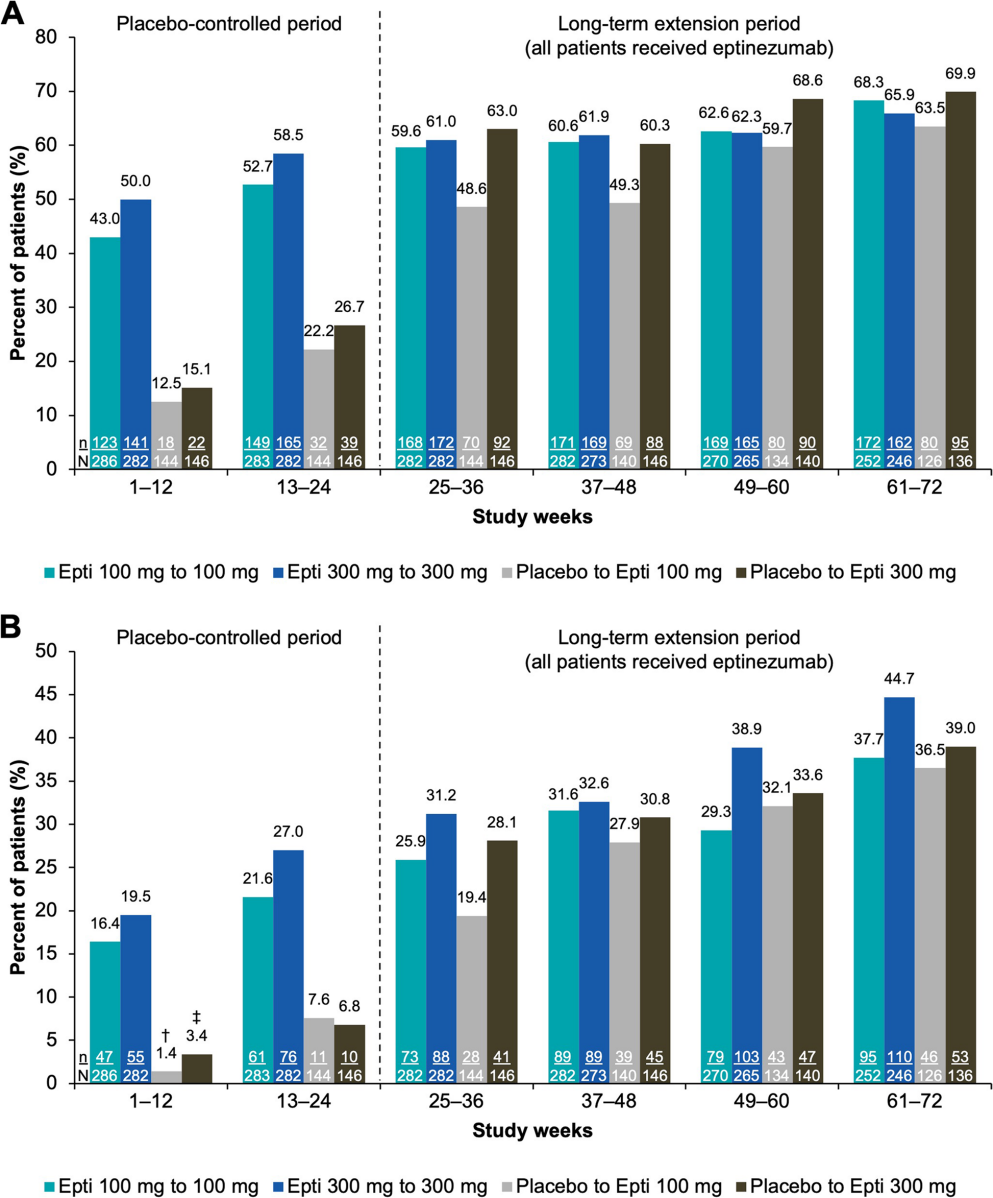
Patients achieving (**A**) ≥ 50% and (**B**) ≥ 75% reduction in monthly migraine days (efficacy analysis set). ^†^n/N = 2/144; ^‡^n/N = 5/146. Epti, eptinezumab

Migraine responder rates are depicted in Fig. 3. For patients who switched from placebo to eptinezumab, the first eptinezumab dose (Weeks 25–36) resulted in a more than doubling of ≥ 50% responder rates and an approximate tripling of ≥ 75% responder rates relative to the preceding dosing interval (Weeks 13–24).

### Headache impact

**Fig. 4:**
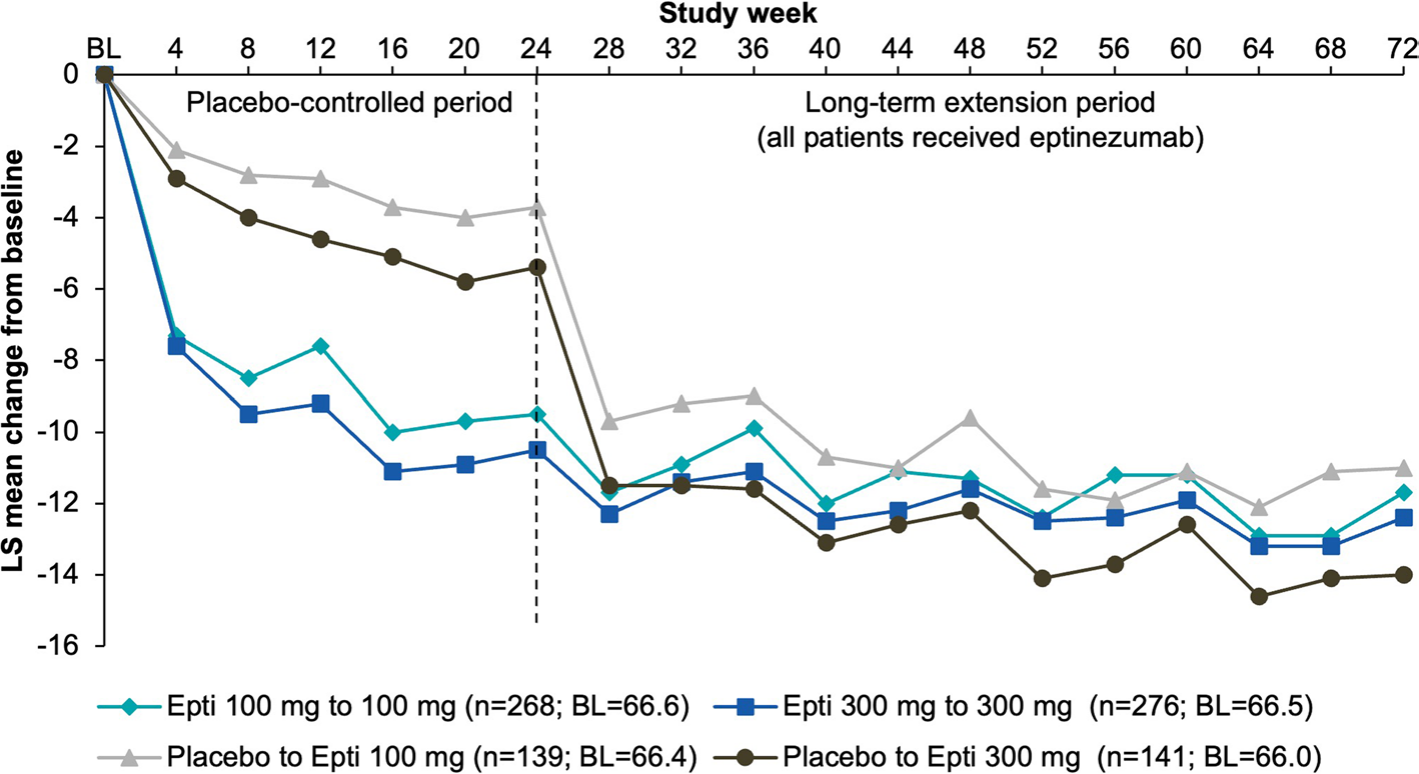
Mean changes from baseline in HIT-6 total score (MMRM; efficacy analysis set). BL, baseline; Epti, eptinezumab; HIT-6, 6-item Headache Impact Test; LS, least squares; MMRM, mixed model for repeated measures

Mean HIT-6 total scores were similar across treatment groups at baseline (66.0–66.6), indicating a population with severe headache-related life impact. Improvement was observed at the first assessment after the first eptinezumab infusion for those initially treated with eptinezumab and for those switching from placebo to eptinezumab for the extension period (Fig. 4). HIT-6 total score was reduced by a mean (SE) of −7.3 (0.56) and −7.6 (0.55) points at Week 4 in patients that received eptinezumab 100 mg and 300 mg, respectively, from the start of the study, and by −9.7 (0.83) and −11.5 (0.83) at Week 28 in patients that were switched to eptinezumab 100 mg and 300 mg, respectively. By the end of the extension, mean HIT-6 total scores were reduced by a similar extent across groups (−11.0 to −14.0 points); this represents a change in headache-related life impact from severe impact (mean HIT-6 total score was ~ 66.4 at baseline across groups; total score range 60–78 = severe) to some impact (mean HIT-6 total score was ~ 52–55 over Weeks 69–72 across groups; total score range 50–55 = some) for these participants.

**Fig. 5:**
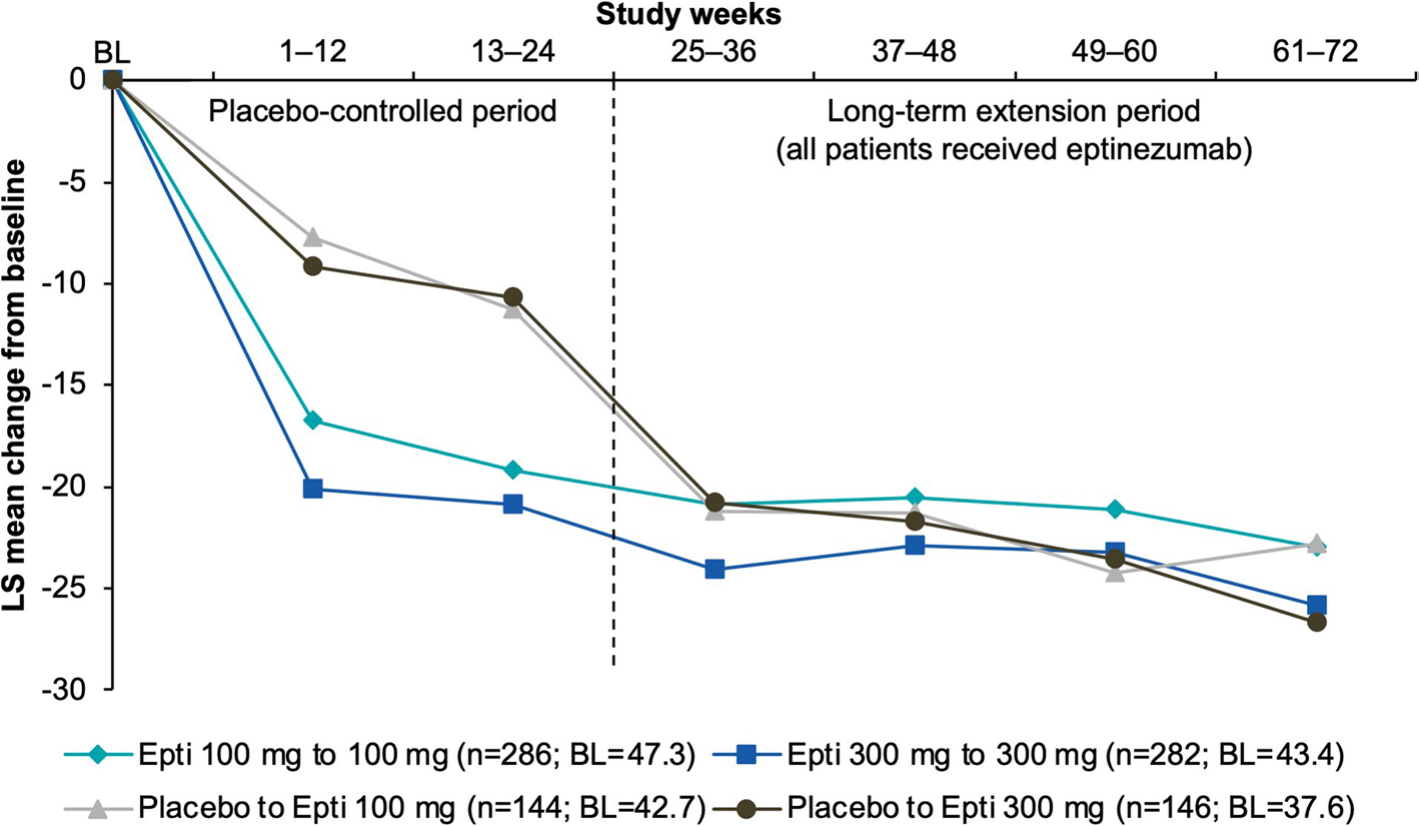
Mean changes from baseline in the percentage of migraine attacks that were severe (MMRM; efficacy analysis set). BL, baseline; Epti, eptinezumab; LS, least squares; MMRM, mixed model for repeated measures

 At baseline, the proportion of migraine attacks with severe intensity ranged 37.6–47.3% across treatment groups. Reductions in severe migraine attacks with eptinezumab were sustained or further improved with continued treatment (Fig. 5). Improvement was observed soon after the start of eptinezumab treatment, with mean (SE) reductions of 18.4% (1.89) and 19.9% (1.89) over Weeks 1–4 in patients initially receiving eptinezumab 100 mg and 300 mg, respectively, and mean (SE) reductions of 21.3% (2.66) and 23.9% (2.67) over Weeks 25–28 in patients switched from placebo to eptinezumab 100 mg and 300 mg, respectively. Over the final 4 weeks (Weeks 69–72), the proportion of migraine attacks reported as severe was reduced by 22.1‒25.3% across treatment groups relative to baseline.

### Acute migraine medication use

**Fig. 6:**
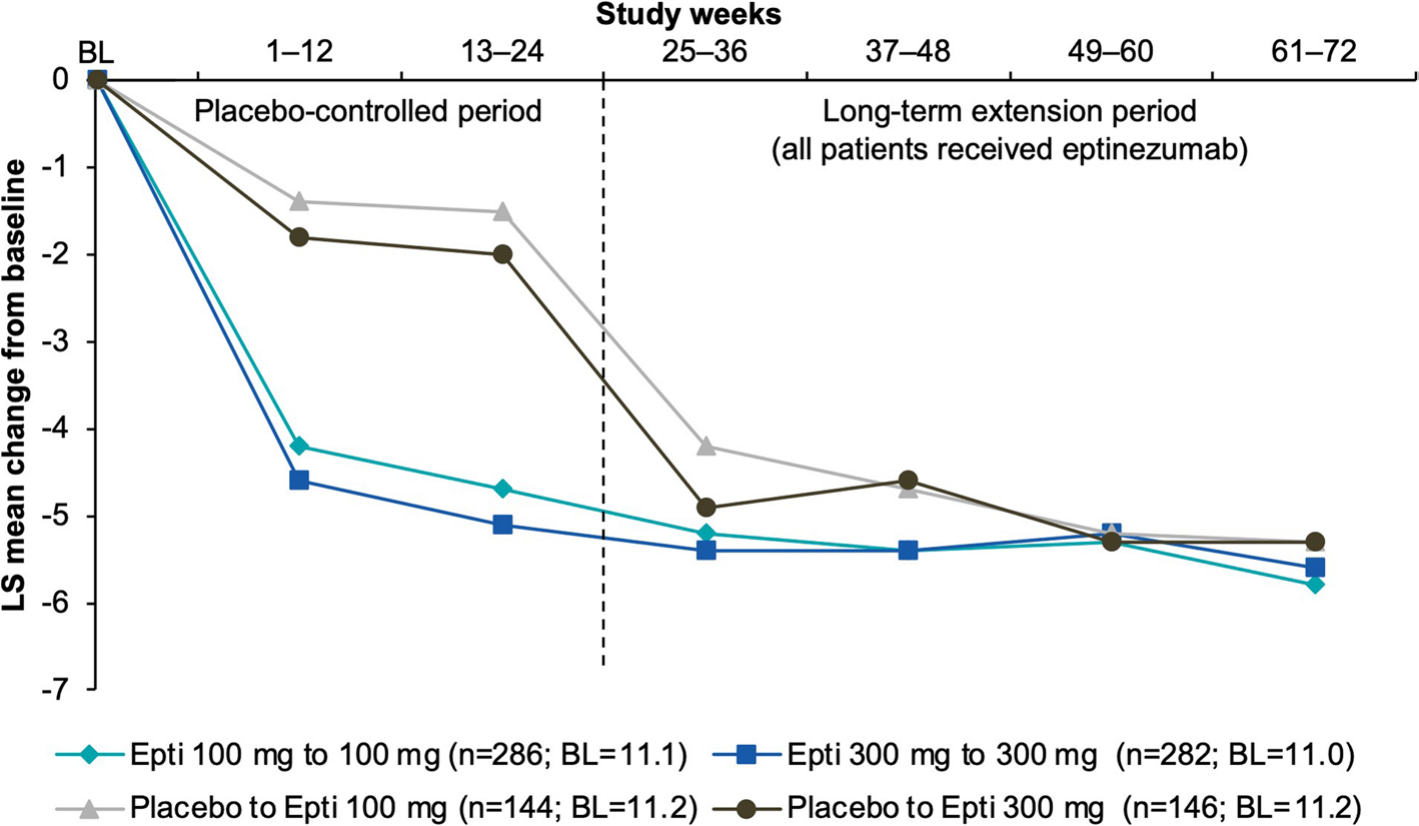
Mean changes from baseline in acute migraine medication days/month (MMRM; efficacy analysis set). Acute migraine medication types included ergotamine, triptan, simple analgesic, combination analgesic, and opioid. BL, baseline; Epti, eptinezumab; LS, least squares; MMRM, mixed model for repeated measures

At baseline, patients across treatment groups were using acute migraine medications for ~ 11 days/month, generally above the ICHD-3 threshold for acute medication overuse, depending on type of acute medication used. In all groups, patients used ~ 5 fewer acute migraine medication days/month during the first eptinezumab dosing interval compared to baseline (Weeks 1–12 for patients initially treated with eptinezumab and Weeks 25–36 for patients switched from placebo to eptinezumab). Thus, the population mean for acute medication use was reduced to below ICHD-3 thresholds for acute medication overuse. Use remained at similarly reduced levels for the remainder of eptinezumab treatment (Fig. 6).

### Other patient-reported outcomes

The impact of treatment on other patient-reported outcomes was also positive, demonstrating consistency with each additional dose, and with the placebo-to-active arms achieving similar outcomes to the active-to-active arms over time (Supplemental Table 1). By the end of the study, mean PI-MBS and PGIC scores reflected “much improvement” in patients’ most bothersome symptom and disease status, respectively (Supplemental Figs. 1 and 2). Mean MSQ role function‒restrictive subscores had improved by 32 to 40 points and role function‒preventive and emotional function subscores by 26–33 points each (Supplemental Table 1). Mean EQ-5D-5L scores had improved by 6–9 points (Supplemental Fig. 3). Working patients reported 3.5‒7.4 fewer days missed (WPAI:M absenteeism), and reductions in WPAI:M presenteeism, work productivity loss, and activity impairment subscores were consistent with greater ability to function at work when present (Supplemental Table 1).

### Safety

Treatment-emergent adverse events (TEAEs) were reported with similar frequency across groups (Table 2); the most commonly reported TEAEs were COVID-19, nasopharyngitis, and upper respiratory tract infection. The proportions of patients with serious adverse events, TEAEs leading to withdrawal, and TEAEs leading to infusion interruption were low.

**Table 2:** Treatment-emergent adverse events in the open-label extension period (safety analysis set)

	No. (%)			
	Epti 100 mg / Epti 100 mg (*n* = 288)	Epti 300 mg / Epti 300 mg (*n* = 284)	Placebo / Epti 100 mg (*n* = 145)	Placebo / Epti 300 mg (*n* = 148)
Any TEAE	159 (55.2)	148 (52.1)	72 (49.7)	81 (54.7)
Any SAE	9 (3.1)	9 (3.2)	2 (1.4)	7 (4.7)
TEAE leading to withdrawal	3 (1.0)	6 (2.1)	0	3 (2.0)
TEAE leading to infusion interruption/termination	1 (0.3)	1 (0.4)	0	0
Death	0	0	0	0
TEAEs in ≥ 1.5% of patients				
COVID-19	63 (21.9)	63 (22.2)	25 (17.2)	31 (20.9)
Nasopharyngitis	19 (6.6)	27 (9.5)	7 (4.8)	13 (8.8)
Upper respiratory tract infection	13 (4.5)	8 (2.8)	4 (2.8)	6 (4.1)
Arthralgia	6 (2.1)	6 (2.1)	1 (0.7)	2 (1.4)
Pruritus	2 (0.7)	6 (2.1)	1 (0.7)	0
Dyspepsia	1 (0.3)	5 (1.8)	0	1 (0.7)
Gastroenteritis	3 (1.0)	5 (1.8)	1 (0.7)	1 (0.7)
Post-vaccination syndrome	0	5 (1.8)	0	1 (0.7)
Sinusitis	4 (1.4)	5 (1.8)	2 (1.4)	2 (1.4)
Urinary tract infection	3 (1.0)	5 (1.8)	3 (2.1)	4 (2.7)
Pharyngitis	3 (1.0)	4 (1.4)	0	3 (2.0)
Upper abdominal pain	4 (1.4)	3 (1.1)	1 (0.7)	3 (2.0)
Bronchitis	4 (1.4)	3 (1.1)	1 (0.7)	3 (2.0)
Cystitis	1 (0.3)	3 (1.1)	0	5 (3.4)
Migraine	7 (2.4)	3 (1.1)	0	1 (0.7)
Nausea	2 (0.7)	3 (1.1)	1 (0.7)	5 (3.4)
Back pain	8 (2.8)	2 (0.7)	1 (0.7)	2 (1.4)
Fatigue	5 (1.7)	2 (0.7)	0	1 (0.7)
Hypertension	3 (1.0)	2 (0.7)	3 (2.1)	0
Menopause	1 (0.3)	0	1 (0.7)	3 (2.0)

*Epti *Eptinezumab, *SAE *Serious adverse event, *TEAE *Treatment-emergent adverse event

## Discussion

The long-term extension phase of the DELIVER trial shows that the benefits of eptinezumab treatment in reducing migraine frequency and impact are sustained for up to 18 months in patients with migraine and 2–4 prior preventive treatment failures. Patients who were originally randomized to placebo during the double-blind phase experienced an immediate decrease in MMDs with the first dose of eptinezumab in the extension phase that was a similar level of MMDs experienced by those who received eptinezumab during the placebo-controlled phase. The improvements observed over time with each additional infusion suggests a sustained or potentially additive effect of continued treatment, possibly justifying continuing eptinezumab beyond current stopping rules. This is especially important in consideration that a sizable portion of the DELIVER population may meet criteria for resistant or refractory migraine [19], which are frequently encountered in clinical practice and for which less than half of healthcare providers are highly confident in treating [20]. Intravenous eptinezumab has also been suggested as a rescue treatment in the emergency department to be then continued as preventative, attesting to long-term benefits [21]. The dose regimens used in DELIVER (100 or 300 mg every 12 weeks) are those currently approved by the US Food and Drug Administration [22] and the European Medicines Agency [23], among other regulatory authorities.

While the migraine responder rates tended to favor the higher eptinezumab dose (300 mg) in the placebo-controlled phase of the study, the effectiveness of the 100-mg dose approached that of the 300-mg dose with longer-term treatment. The proportion of patients experiencing a ≥ 50% reduction in MMDs during the final 12 weeks of the long-term extension was similar across all 4 treatment groups. The ≥ 75% migraine responder rates, however, continued to favor the higher dose at this timepoint. At present, long-term data beyond 1 year in similar patient populations are available for only one other anti-CGRP monoclonal antibody (erenumab) [24]; data from other agents are limited to 3–6 months [1, 7]. The proportions of patients achieving a ≥ 50% reduction in MMDs in the 2-year LIBERTY study were 47% over Weeks 61–64 and 57% over Weeks 109–112; ≥75% responder rates were 23% and 31% over Weeks 61–64 and 109–112, respectively [24].

Following eptinezumab initiation, patients used ~ 5 fewer days/month of acute migraine medications use when compared to baseline, bringing the average to below thresholds for medication overuse [17]. The change from baseline in patients switching from placebo to eptinezumab was similar in magnitude to that observed in patients initially randomized to eptinezumab, and across groups, acute medication use remained at a similarly reduced level for the remainder of eptinezumab treatment. This decrease in the amount of acute migraine medication needed signifies the long-term effectiveness of eptinezumab and its potential to revert and/or prevent medication overuse in a refractory patient population [25].

Improvements in patient-reported outcomes indicate that patients perceived benefits beyond reduced migraine frequency and acute medication use with eptinezumab therapy. Across measures of disease-related functioning, impact, quality of life, and work productivity, patients treated with eptinezumab experienced improvements following the first administration, which demonstrated consistency or further improvement with each subsequent administration. By the end of study, scores indicated that the average patient in DELIVER shifted from experiencing severe headache-related life impact to moderate impact (HIT-6 total score), had clinically meaningful improvement in health-related quality of life (MSQ subscores), experienced much improvement in their most bothersome symptom (PI-MBS) and overall disease state (PGIC), and reported increased work productivity (WPAI:M). These findings are consistent with those of previous eptinezumab studies, which have demonstrated long-term reductions in disability and improvements in functioning and quality of life over periods of up to 2 years (PROMISE-1 [9], PROMISE-2 [11], PREVAIL [26]).

In this long-term study of eptinezumab, there was a low dropout rate, indicating tolerability, and TEAEs with an incidence of ≥ 1.5% were of similar nature to those most commonly reported in previous studies [4, 8–13]. Other phase 3b studies with anti-CGRP monoclonal antibodies in similar populations have also demonstrated high completion rates over shorter open-label extension studies. The completion rate in this study was 90.4% and consistent with findings from the LIBERTY study, in which 85% of patients completed 13 months of an open-label extension of the original 3-month double-blind study and 75% completed 25 months [24, 27]. Long-term treatment with eptinezumab in the real-world may improve persistence/adherence to a preventive medication and thereby lower burden/disability, improve function, and reduce presenteeism/absenteeism.

### Limitations

The extension phase of the DELIVER trial lacked a placebo or active comparator arm, which may limit the interpretation of the results; however, dose-blinding was maintained for patients and investigators (but not for the sponsor) throughout the extension phase. Although the study was primarily conducted in Europe, the results are likely applicable to the US. Enrolled patients were predominantly White and female, limiting generalizability. Additional studies in broader patient populations are needed to confirm that observed benefits extend to these populations. DELIVER did, however, include older participants (up to and including 75 years of age) and those with comorbidities that were excluded in previous trials. The inclusion of these patients did not appear to be associated with any new safety concerns. Lastly, DELIVER did not allow for dose escalation or reduction; thus, the effects of changing dose levels during long-term treatment cannot be evaluated.

## Conclusion

This long-term extension study highlights eptinezumab as an effective and well-tolerated long-term preventive migraine treatment option for adults with a history of 2–4 prior preventive treatment failures. This study had high completion rates and demonstrated tolerability and benefits sustained for up to 18 months across migraine endpoints, including reductions in frequency and severity of migraine, reductions in acute medication use, high responder rates, and patient-reported improvements in functioning, disease status, and health-related quality of life.

### Supplementary Information

**Additional file 1: Supplemental Methods.** Patient-reported outcomes. **Supplemental Table 1. **Monthly migraine days and patient-reported outcomes (MMRM; efficacy analysis set). **Supplemental Figure 1. **Mean PI-MBS score (MMRM; efficacy analysis set). **Supplemental Figure 2. **Mean PGIC score (MMRM; efficacy analysis set). **Supplemental Figure 3. **Mean change from baseline in EQ-5D-5L VAS score (MMRM; efficacy analysis set).

## Data Availability

In accordance with EFPIA’s and PhRMA’s “Principles for Responsible Clinical Trial Data Sharing” guidelines, Lundbeck is committed to responsible sharing of clinical trial data in a manner that is consistent with safeguarding the privacy of patients, respecting the integrity of national regulatory systems, and protecting the intellectual property of the sponsor. The protection of intellectual property ensures continued research and innovation in the pharmaceutical industry. Deidentified data are available to those whose request has been reviewed and approved through an application submitted to https://www.lundbeck.com/global/our-science/clinical-data-sharing.

## Abbreviations

CGRP: Calcitonin gene-related peptide
HIT-6: 6-item Headache Impact Test
MHDs: Monthly headache days
MMDs: Monthly migraine days
MSQ: Migraine-Specific Quality of Life questionnaire
PGIC: Patient Global Impression of Change
PI-MBS: Patient-identified most bothersome symptom
TEAEs: Treatment-emergent adverse events
WPAI:M: Work Productivity and Impairment, adapted for Migraine
