# Supplementary material for: Long-term effectiveness of eptinezumab in patients with migraine and prior preventive treatment failures: extension of a randomized controlled trial
Source: J Headache Pain. 2023 Nov 20;24(1):155. doi: 10.1186/s10194-023-01688-w (PMC10662788; doi:10.1186/s10194-023-01688-w)
Supplement: Supplementary file 1 — Additional file 1: Supplemental Methods. Patient-reported outcomes. Supplemental Table 1. Monthly migraine days and patient-reported outcomes (MMRM; efficacy analysis set). Supplemental Figure 1. Mean PI-MBS score (MMRM; efficacy analysis set). Supplemental Figure 2. Mean PGIC score (MMRM; efficacy analysis set). Supplemental Figure 3. Mean change from baseline in EQ-5D-5L VAS score (MMRM; efficacy analysis set). [file 10194_2023_1688_MOESM1_ESM.pdf]

Online Supplemental Material

Supplemental Methods. Patient-reported outcomes

Supplemental Table 1. Monthly migraine days and patient-reported outcomes (MMRM; efficacy analysis set)

Supplemental Figure 1. Mean PI-MBS score (MMRM; efficacy analysis set)

Supplemental Figure 2. Mean PGIC score (MMRM; efficacy analysis set)

Supplemental Figure 3. Mean change from baseline in EQ-5D-5L VAS score (MMRM; efficacy analysis set)

Supplemental Methods. Patient-reported outcomes

Headache Impact Test (HIT-6)

The HIT-6 comprises 6 items (severe pain, activity limitations [household, work, school, or social], wish you could lie down, too tired [4-week recall], fed up/irritated [4-week recall], and ability to concentrate [4-week recall]) rated using a Likert-type scale. The HIT-6 total score can range from 36 to 78. Total score reductions within-person of 2.5 to 6 points or more are considered clinically meaningful.[1,2]

Patient-Identified Most Bothersome Symptom (PI-MBS)

Patients verbally identified their most bothersome symptom at screening and rated their perceived level of improvement at each subsequent visit using a 7-point scale (1=very much improved, 2=much improved, 3=minimally improved, 4=no change, 5=minimally worse, 6=much worse, and 7=very much worse).[3]

Patient Global Impression of Change (PGIC)

The PGIC comprises a single question regarding the patient's impression of the change in their disease status since the start of the study. Patients rated their perceived level of improvement at each visit using a 7-point scale (1=very much improved, 2=much improved, 3=minimally improved, 4=no change, 5=minimally worse, 6=much worse, and 7=very much worse).[4]

Migraine-Specific Quality of Life questionnaire (MSQ)

The MSQ is a patient-reported outcome designed to assess the quality of life in patients with migraine. It comprises 14 items covering 3 domains: role function restrictive (7 items); role

function preventive (4 items); and emotional function (3 items). Patients rated each item on a 6-point scale ranging from 1 (none of the time) to 6 (all of the time). Raw domain scores were summed and transformed to a 0- to 100-point scale. Higher scores are indicative of better quality of life.[5]

EQ-5D-5L

The EQ-5D-5L comprises 5 descriptive items (mobility, self-care, usual activities, pain/discomfort, and depression/anxiety) and a VAS of the overall health state. The VAS ranges from 0 (worst imaginable health state) to 100 (best imaginable health state).[6,7]

Work Productivity Activity Index adapted for Migraine (WPAI:M)

The WPAI:M comprises 6 questions that assess activities over the preceding 7 days. The first question addresses whether the patient is currently employed. The subsequent 3 questions assess the number of hours missed from work because of problems with migraine, how many hours that were missed from work due to any other reason, and the number of hours the patient actually worked. The last 2 questions are VAS that assess how much the migraine affected productivity while working, and how much it affected the ability to perform regular daily activities, other than work at a job. Responses were used to derive 4 subscores: absenteeism, work productivity, presenteeism, and activity impairment.[8]

References

1. Houts CR, Wirth RJ, McGinley JS, Cady R, Lipton RB. Determining thresholds for meaningful change for the Headache Impact Test (HIT-6) total and item-specific scores in chronic migraine. *Headache*. 2020;60(9):2003-2013. doi:10.1111/head.13946
2. Smelt AF, Assendelft WJ, Terwee CB, Ferrari MD, Blom JW. What is a clinically relevant change on the HIT-6 questionnaire? An estimation in a primary-care population of migraine patients. Cephalalgia. 2014 Jan;34(1):29-36. doi: 10.1177/0333102413497599.
3. Lipton RB, Dodick DW, Ailani J, McGill L, Hirman J, Cady R. Patient-identified most bothersome symptom in preventive migraine treatment with eptinezumab: a novel patient-centered outcome. Headache. May 2021;61(5):766-776. doi:10.1111/head.14120
4. Guy W. ECDEU Assessment Manual for Psychopharmacology. U.S. Department of Health, Education, and Welfare, Public Health Service, Alcohol, Drug Abuse, and Mental Health Administration, National Institute of Mental Health, Psychopharmacology Research Branch, Division of Extramural Research Programs; 1976
5. Jhingran P, Osterhaus JT, Miller DW, Lee JT, Kirchdoerfer L. Development and validation of the Migraine-Specific Quality of Life Questionnaire. *Headache*. Apr 1998;38(4):295-302. doi:10.1046/j.1526-4610.1998.3804295.x
6. Rabin R, de Charro F. EQ-5D: a measure of health status from the EuroQol Group. Ann Med. Jul 2001;33(5):337-43. doi:10.3109/07853890109002087
7. Herdman M, Gudex C, Lloyd A, et al. Development and preliminary testing of the new five-level version of EQ-5D (EQ-5D-5L). Qual Life Res. Dec 2011;20(10):1727-36. doi:10.1007/s11136-011-9903-x

8. Reilly MC, Zbrozek AS, Dukes EM. The validity and reproducibility of a work productivity and activity impairment instrument. Pharmacoeconomics. Nov 1993;4(5):353-65. doi:10.2165/00019053-199304050-00006

Supplemental Table 1. Monthly migraine days and patient-reported outcomes (MMRM; efficacy analysis set)

	Study week(s)	Epti 100 mg / Epti 100 mg	Epti 300 mg / Epti 300 mg	Placebo / Epti 100 mg	Placebo / Epti 300 mg
**Monthly migraine days**					
Change from BL, mean (SE)	1–4	−5.3 (0.36)	−5.7 (0.36)	−1.7 (0.45)	−2.1 (0.45)
	5–8	−5.3 (0.37)	−5.7 (0.37)	−2.4 (0.47)	−3.0 (0.47)
	9–12	−4.6 (0.38)	−5.0 (0.38)	−2.0 (0.49)	−2.7 (0.49)
	13–16	−6.0 (0.39)	−6.6 (0.38)	−2.0 (0.49)	−3.2 (0.49)
	17–20	−5.5 (0.39)	−6.3 (0.39)	−2.3 (0.50)	−3.2 (0.49)
	21–24	−5.6 (0.39)	−5.7 (0.39)	−2.3 (0.50)	−2.8 (0.50)
	25–28	−7.2 (0.39)	−6.9 (0.39)	−5.8 (0.50)	−7.2 (0.50)
	29–32	−6.3 (0.39)	−6.4 (0.39)	−5.1 (0.50)	−6.6 (0.50)
	33–36	−5.4 (0.40)	−5.9 (0.40)	−4.8 (0.52)	−6.1 (0.52)
	37–40	−7.0 (0.40)	−6.8 (0.40)	−6.0 (0.52)	−6.9 (0.52)
	41–44	−6.2 (0.41)	−6.9 (0.41)	−5.4 (0.52)	−6.6 (0.52)
	45–48	−5.6 (0.41)	−6.0 (0.41)	−5.1 (0.53)	−6.0 (0.53)
	49–52	−6.8 (0.40)	−6.8 (0.40)	−6.6 (0.52)	−7.5 (0.52)
	53–56	−6.3 (0.40)	−6.7 (0.40)	−6.4 (0.52)	−7.3 (0.52)
	57–60	−5.7 (0.42)	−6.1 (0.42)	−5.5 (0.55)	−6.4 (0.54)
	61–64	−7.1 (0.42)	−7.0 (0.42)	−7.0 (0.54)	−7.8 (0.53)
	65–68	−7.3 (0.41)	−7.1 (0.41)	−6.4 (0.53)	−7.2 (0.52)
	69–72	−6.8 (0.41)	−6.9 (0.41)	−5.9 (0.53)	−6.8 (0.52)
**HIT-6 total score**					
Change from BL, mean (SE)	4	−7.3 (0.56)	−7.6 (0.55)	−2.1 (0.70)	−2.9 (0.71)
	8	−8.5 (0.60)	−9.5 (0.59)	−2.8 (0.76)	−4.0 (0.77)
	12	−7.6 (0.58)	−9.2 (0.57)	−2.9 (0.73)	−4.6 (0.73)
	16	−10.0 (0.61)	−11.1 (0.60)	−3.7 (0.77)	−5.1 (0.78)
	20	−9.7 (0.61)	−10.9 (0.60)	−4.0 (0.77)	−5.8 (0.78)
	24	−9.5 (0.59)	−10.5 (0.58)	−3.7 (0.75)	−5.4 (0.75)
	28	−11.7 (0.65)	−12.3 (0.64)	−9.7 (0.83)	−11.5 (0.83)
	32	−10.9 (0.62)	−11.4 (0.61)	−9.2 (0.79)	−11.5 (0.80)
	36	−9.9 (0.62)	−11.1 (0.61)	−9.0 (0.79)	−11.6 (0.79)
	40	−12.0 (0.64)	−12.5 (0.63)	−10.7 (0.81)	−13.1 (0.81)
	44	−11.1 (0.64)	−12.2 (0.63)	−11.0 (0.82)	−12.6 (0.82)
	48	−11.3 (0.65)	−11.6 (0.64)	−9.6 (0.83)	−12.2 (0.83)
	52	−12.4 (0.65)	−12.5 (0.64)	−11.6 (0.83)	−14.1 (0.84)
	56	−11.2 (0.66)	−12.4 (0.64)	−11.9 (0.84)	−13.7 (0.80)
	60	−11.2 (0.63)	−11.9 (0.62)	−11.1 (0.80)	−12.6 (0.83)
	64	−12.9 (0.65)	−13.2 (0.64)	−12.1 (0.83)	−14.6 (0.82)
	68	−12.9 (0.64)	−13.2 (0.63)	−11.1 (0.82)	−14.1 (0.82)
	72	−11.7 (0.66)	−12.4 (0.65)	−11.0 (0.84)	−14.0 (0.84)
**PI-MBS score**[a]					
n/mean (SE)	12	280/2.8 (0.08)	279/2.6 (0.08)	139/3.7 (0.10)	146/3.6 (0.10)
	24	272/2.7 (0.08)	275/2.5 (0.08)	136/3.8 (0.10)	142/3.4 (0.10)
	36	277/2.6 (0.08)	276/2.5 (0.08)	138/2.7 (0.10)	142/2.5 (0.10)
	48	273/2.5 (0.08)	264/2.4 (0.08)	134/2.7 (0.10)	144/2.5 (0.10)
	60	261/2.4 (0.08)	262/2.3 (0.08)	128/2.6 (0.10)	141/2.3 (0.10)

	Study week(s)	Epti 100 mg / Epti 100 mg	Epti 300 mg / Epti 300 mg	Placebo / Epti 100 mg	Placebo / Epti 300 mg
	72	250/2.3 (0.08)	241/2.3 (0.08)	124/2.5 (0.10)	136/2.1 (0.10)
**PGIC score[a]**					
n/mean (SD)	4	272/2.5 (0.08)	271/2.4 (0.08)	141/3.6 (0.10)	135/3.4 (0.10)
	12	283/2.5 (0.08)	281/2.4 (0.08)	143/3.6 (0.10)	146/3.5 (0.10)
	24	275/2.5 (0.08)	277/2.3 (0.08)	140/3.6 (0.10)	142/3.3 (0.10)
	36	281/2.4 (0.08)	278/2.2 (0.08)	142/2.6 (0.10)	142/2.3 (0.10)
	48	277/2.3 (0.08)	266/2.1 (0.08)	139/2.5 (0.10)	144/2.1 (0.10)
	60	265/2.1 (0.07)	264/2.1 (0.07)	133/2.3 (0.09)	141/2.1 (0.09)
	72	254/2.1 (0.07)	243/2.0 (0.07)	128/2.2 (0.09)	136/1.9 (0.09)
**MSQ role function-restrictive subscore**					
Baseline, n/mean	BL	263/35.6	276/35.7	139/35.6	141/35.4
Change from baseline, n/LS mean (SE)	12	262/26.2 (1.56)	275/30.0 (1.52)	139/12.1 (1.94)	141/17.2 (1.95)
	24	254/31.4 (1.57)	271/31.4 (1.52)	136/13.0 (1.95)	138/18.9 (1.95)
	36	259/30.9 (1.57)	272/33.5 (1.53)	138/29.5 (1.96)	137/35.2 (1.97)
	48	255/32.2 (1.57)	260/34.9 (1.54)	134/30.3 (1.96)	139/35.4 (1.96)
	60	244/34.0 (1.53)	258/34.8 (1.49)	128/32.3 (1.90)	136/37.3 (1.89)
	72	234/34.6 (1.57)	237/36.5 (1.54)	124/32.1 (1.96)	131/39.7 (1.94)
**MSQ role function-preventive subscore**					
Baseline, n/mean	BL	263/50.3	276/51.3	139/51.4	141/49.8
Change from baseline, n/LS mean (SE)	12	262/24.3 (1.45)	275/26.8 (1.42)	139/11.0 (1.82)	141/15.2 (1.82)
	24	254/27.3 (1.44)	271/28.1 (1.40)	136/11.4 (1.80)	138/17.9 (1.80)
	36	259/26.9 (1.42)	272/28.9 (1.38)	138/25.0 (1.76)	137/31.3 (1.77)
	48	255/27.5 (1.41)	260/30.1 (1.38)	134/25.8 (1.76)	139/30.4 (1.75)
	60	244/28.9 (1.38)	258/30.0 (1.34)	128/27.9 (1.71)	136/31.2 (1.69)
	72	234/29.5 (1.44)	237/30.4 (1.41)	124/26.3 (1.79)	131/33.0 (1.77)
**MSQ emotional function subscore**					
Baseline, n/mean	BL	263/50.6	276/48.4	139/48.4	141/49.0
Change from baseline, n/LS mean (SE)	12	262/23.5 (1.65)	275/26.2 (1.60)	139/9.7 (2.05)	141/14.9 (2.05)
	24	254/26.8 (1.65)	271/27.1 (1.60)	136/8.8 (2.05)	138/16.6 (2.05)
	36	259/25.9 (1.64)	272/28.2 (1.59)	138/24.3 (2.03)	137/29.9 (2.04)
	48	255/27.9 (1.63)	260/29.3 (1.59)	134/25.0 (2.02)	139/30.0 (2.01)
	60	244/28.7 (1.59)	258/29.2 (1.55)	128/27.3 (1.97)	136/30.2 (1.96)
	72	234/28.1 (1.63)	237/30.2 (1.60)	124/25.5 (2.02)	131/33.4 (2.00)
**EQ-5D-5L VAS score**					
Baseline, n/mean	BL	263/76.0	274/74.6	138/75.4	141/72.6
Change from baseline, n/LS mean (SE)	4	254/2.6 (1.26)	265/2.4 (1.23)	136/−4.6 (1.58)	132/−0.4 (1.61)
	8	258/1.3 (1.35)	267/4.1 (1.32)	134/−4.3 (1.74)	136/−0.8 (1.74)
	12	262/3.1 (1.31)	273/4.9 (1.28)	138/−2.5 (1.67)	141/−2.7 (1.66)
	16	257/3.7 (1.32)	265/4.9 (1.29)	136/−2.0 (1.69)	135/−1.5 (1.69)
	20	251/3.5 (1.37)	266/2.9 (1.33)	134/−4.5 (1.75)	135/−1.4 (1.75)
	24	253/2.7 (1.29)	269/5.6 (1.25)	135/−2.3 (1.63)	137/−2.0 (1.63)
	28	254/5.2 (1.30)	270/5.5 (1.26)	134/2.8 (1.65)	138/6.3 (1.65)
	32	256/4.1 (1.29)	269/4.9 (1.26)	133/0.7 (1.64)	134/5.6 (1.64)
	36	260/5.4 (1.25)	270/6.1 (1.23)	137/4.1 (1.59)	137/6.5 (1.59)
	40	249/5.7 (1.32)	254/4.1 (1.30)	128/4.0 (1.70)	135/6.3 (1.68)
	44	250/5.8 (1.29)	258/3.5 (1.26)	127/5.5 (1.65)	135/5.8 (1.63)
	48	255/4.6 (1.34)	258/4.4 (1.32)	133/1.5 (1.72)	139/4.8 (1.71)
	52	240/4.4 (1.29)	246/4.9 (1.26)	125/6.7 (1.64)	132/7.9 (1.62)
	56	236/4.6 (1.34)	244/4.2 (1.31)	126/5.6 (1.70)	129/8.3 (1.69)

	Study week(s)	Epti 100 mg / Epti 100 mg	Epti 300 mg / Epti 300 mg	Placebo / Epti 100 mg	Placebo / Epti 300 mg
	60	244/7.2 (1.28)	256/5.2 (1.25)	127/6.1 (1.63)	136/7.2 (1.61)
	64	227/6.3 (1.33)	231/4.9 (1.31)	120/7.9 (1.70)	122/6.4 (1.69)
	68	233/5.5 (1.32)	231/6.2 (1.30)	122/5.9 (1.69)	127/7.9 (1.67)
	72	234/6.8 (1.25)	234/6.6 (1.23)	124/6.6 (1.57)	131/8.9 (1.56)
**WPAI:M absenteeism subscore**					
Baseline, n/mean	BL	187/11.8	198/11.7	102/10.7	106/14.4
Change from baseline, n/LS mean (SE)	4	161/−6.4 (1.12)	177/−7.7 (1.06)	90/−3.7 (1.40)	97/−0.4 (1.38)
	8	172/−5.9 (1.40)	180/−4.4 (1.35)	86/−0.8 (1.86)	93/−0.8 (1.81)
	12	170/−5.8 (1.41)	178/−4.2 (1.37)	95/−0.1 (1.82)	94/−0.3 (1.83)
	16	152/−5.8 (1.29)	166/−6.3 (1.24)	81/−2.7 (1.68)	83/0.6 (1.67)
	20	156/−4.2 (1.50)	158/−4.2 (1.47)	83/−1.4 (1.97)	80/2.2 (2.00)
	24	148/−5.1 (1.39)	167/−5.5 (1.31)	83/0.1 (1.78)	93/−1.9 (1.72)
	28	153/−7.0 (1.31)	161/−4.8 (1.26)	83/−5.4 (1.67)	95/−7.5 (1.61)
	32	163/−5.5 (1.18)	173/−5.3 (1.13)	80/−7.0 (1.55)	82/−6.9 (1.55)
	36	161/−3.6 (1.40)	174/−4.6 (1.34)	88/−3.6 (1.81)	88/−5.5 (1.81)
	40	149/−6.2 (1.42)	159/−4.7 (1.36)	77/−3.7 (1.88)	79/−4.9 (1.86)
	44	146/−4.7 (1.50)	159/−5.6 (1.43)	76/−5.9 (1.98)	86/−1.9 (1.90)
	48	160/−4.3 (1.39)	164/−4.4 (1.35)	82/−3.2 (1.84)	86/−5.7 (1.81)
	52	152/−5.9 (1.32)	150/−4.7 (1.30)	76/−6.5 (1.75)	81/−5.1 (1.71)
	56	143/−4.4 (1.32)	150/−5.5 (1.27)	76/−7.0 (1.69)	82/−5.5 (1.66)
	60	148/−3.5 (1.49)	156/−4.2 (1.44)	84/−4.5 (1.91)	82/−3.5 (1.93)
	64	138/−4.3 (1.34)	131/−6.0 (1.35)	77/−7.1 (1.71)	77/−5.6 (1.72)
	68	135/−4.4 (1.50	129/−6.1 (1.52)	72/−3.1 (1.97)	74/−7.4 (1.96)
	72	138/−5.6 (1.36)	127/−4.8 (1.38)	68/−3.5 (1.81)	72/−7.2 (1.78)
**WPAI:M presenteeism subscore**					
Baseline, n/mean	BL	182/50.8	196/53.5	100/49.7	102/53.3
Change from baseline, n/LS mean (SE)	4	158/−26.2 (2.31)	176/−24.5 (2.17)	89/−9.0 (2.84)	93/−13.0 (2.83)
	8	169/−25.3 (2.33)	176/−23.8 (2.23)	85/−7.3 (2.96)	87/−11.0 (2.97)
	12	165/−20.4 (2.30)	174/−24.7 (2.20)	93/−10.4 (2.83)	88/−11.7 (2.89)
	16	148/−23.3 (2.38)	163/−24.6 (2.26)	79/−2.8 (3.00)	80/−9.5 (3.00)
	20	149/−24.6 (2.40)	156/−22.9 (2.30)	81/−7.3 (3.01)	75/−13.4 (3.07)
	24	142/−23.6 (2.39)	165/−20.7 (2.24)	79/−8.2 (2.97)	90/−10.3 (2.90)
	28	147/−27.7 (2.38)	158/−23.4 (2.27)	80/−25.9 (2.98)	92/−27.1 (2.89)
	32	157/−21.9 (2.33)	170/−23.1 (2.20)	79/−24.4 (2.96)	80/−25.0 (2.97)
	36	153/−24.2 (2.30)	172/−22.3 (2.17)	86/−20.9 (2.85)	87/−24.2 (2.85)
	40	144/−28.8 (2.27)	155/−25.2 (2.16)	74/−22.6 (2.87)	76/−25.8 (2.85)
	44	140/−23.8 (2.40)	155/−25.7 (2.28)	73/−23.5 (3.05)	83/−22.2 (2.97)
	48	154/−24.2 (2.36)	161/−24.2 (2.26)	81/−22.9 (2.97)	83/−21.5 (2.96)
	52	149/−26.2 (2.30)	145/−23.8 (2.23)	75/−24.6 (2.91)	78/−28.1 (2.90)
	56	138/−24.0 (2.40)	149/−23.8 (2.29)	75/−26.0 (3.01)	79/−27.6 (2.98)
	60	141/−24.7 (2.37)	153/−23.8 (2.25)	81/−22.6 (2.94)	80/−23.4 (2.94)
	64	134/−28.2 (2.36)	130/−25.9 (2.29)	75/−23.7 (2.93)	73/−29.4 (2.95)
	68	127/−28.1 (2.34)	127/−23.9 (2.28)	70/−22.8 (2.92)	71/−30.2 (2.92)
	72	134/−26.0 (2.34)	126/−24.5 (2.28)	66/−24.9 (2.98)	69/−28.4 (2.95)
**WPAI:M work productivity loss subscore**					
Baseline, n/mean	BL	182/53.8	196/57.2	100/52.8	102/57.7
Change from baseline, n/LS mean (SE)	4	158/−26.7 (2.41)	176/−26.1 (2.27)	89/−9.2 (2.96)	93/−12.2 (2.96)
	8	169/−25.6 (2.46)	176/−24.6 (2.36)	85/−6.9 (3.13)	87/−12.0 (3.14)
	12	165/−21.0 (2.42)	174/−25.5 (2.31)	93/−10.0 (2.98)	88/−11.8 (3.04)

	Study week(s)	Epti 100 mg / Epti 100 mg	Epti 300 mg / Epti 300 mg	Placebo / Epti 100 mg	Placebo / Epti 300 mg
	16	148/−24.1 (2.50)	163/−26.1 (2.37)	79/−3.3 (3.14)	80/−9.0 (3.15)
	20	149/−24.8 (2.54)	156/−23.7 (2.44)	81/−7.6 (3.20)	75/−13.6 (3.27)
	24	142/−24.1 (2.51)	165/−21.4 (2.35)	79/−7.8 (3.13)	90/−10.4 (3.05)
	28	147/−28.8 (2.49)	158/−24.5 (2.37)	80/−27.2 (3.12)	92/−29.0 (3.02)
	32	157/−22.2 (2.46)	170/−23.5 (2.33)	79/−25.5 (3.14)	80/−27.0 (3.15)
	36	153/−23.8 (2.47)	172/−22.4 (2.33)	86/−20.5 (3.06)	87/−25.3 (3.06)
	40	144/−29.3 (2.44)	155/−25.8 (2.33)	74/−22.7 (3.11)	76/−27.3 (3.09)
	44	140/−24.6 (2.57)	155/−27.0 (2.44)	73/−24.8 (3.28)	83/−22.5 (3.18)
	48	154/−24.5 (2.52)	161/−24.5 (2.42)	81/−21.4 (3.19)	83/−23.0 (3.18)
	52	149/−26.8 (2.43)	145/−25.1 (2.37)	75/−25.6 (3.09)	78/−28.8 (3.07)
	56	138/−24.4 (2.53)	149/−25.1 (2.41)	75/−27.1 (3.17)	79/−29.2 (3.14)
	60	141/−24.8 (2.55)	153/−24.5 (2.43)	81/−23.4 (3.17)	80/−23.7 (3.18)
	64	134/−27.3 (2.51)	130/−26.5 (2.45)	75/−24.7 (3.13)	73/−30.8 (3.15)
	68	127/−28.8 (2.50)	127/−25.2 (2.43)	70/−21.0 (3.12)	71/−32.2 (3.12)
	72	134/−26.5 (2.50)	126/−25.3 (2.45)	66/−22.9 (3.20)	69/−30.6 (3.17)
**WPAI:M activity impairment subscore**					
Baseline, n/mean	BL	261/58.3	274/58.9	138/58.2	140/59.0
Change from baseline, n/LS mean (SE)	4	251/−23.7 (1.98)	264/−24.5 (1.93)	135/−11.0 (2.49)	132/−12.2 (2.52)
	8	256/−24.5 (2.01)	267/−24.9 (1.96)	134/−11.1 (2.56)	135/−14.4 (2.57)
	12	259/−23.1 (1.91)	273/−25.2 (1.86)	138/−10.6 (2.40)	140/−14.2 (2.40)
	16	253/−26.6 (1.95)	265/−27.6 (1.90)	136/−9.1 (2.45)	134/−13.8 (2.47)
	20	249/−25.6 (1.99)	266/−25.6 (1.93)	134/−10.9 (2.52)	134/−16.3 (2.53)
	24	251/−26.3 (1.90)	269/−23.9 (1.84)	135/−8.3 (2.37)	136/−14.5 (2.38)
	28	252/−29.2 (1.99)	270/−29.6 (1.93)	134/−27.9 (2.52)	137/−31.7 (2.52)
	32	255/−25.5 (1.94)	269/−27.2 (1.89)	133/−25.3 (2.46)	134/−28.5 (2.46)
	36	258/−25.1 (1.94)	270/−26.1 (1.89)	137/−23.3 (2.45)	136/−27.6 (2.46)
	40	247/−29.4 (1.94)	254/−29.5 (1.89)	128/−26.5 (2.45)	133/−29.9 (2.44)
	44	247/−27.9 (1.96)	257/−29.6 (1.91)	127/−25.6 (2.50)	134/−28.2 (2.47)
	48	254/−26.4 (1.94)	258/−28.1 (1.90)	133/−24.5 (2.46)	138/−26.0 (2.45)
	52	239/−28.9 (1.96)	246/−28.4 (1.91)	124/−28.5 (2.48)	131/−33.5 (2.46)
	56	234/−25.8 (1.97)	244/−28.7 (1.92)	126/−29.3 (2.49)	128/−32.0 (2.48)
	60	242/−28.1 (1.98)	256/−27.4 (1.93)	127/−25.6 (2.51)	135/−27.5 (2.48)
	64	225/−30.0 (1.97)	230/−30.9 (1.93)	119/−29.5 (2.50)	121/−32.2 (2.49)
	68	230/−28.7 (1.98)	231/−29.1 (1.95)	122/−26.7 (2.51)	125/−32.6 (2.50)
	72	232/−28.9 (1.97)	233/−28.8 (1.94)	124/−29.9 (2.49)	129/−31.3 (2.48)

[a]Rated on a 7-point scale where 1=very much improved, 2=much improved, 3=minimally improved, 4=no change, 5=minimally worse, 6=much worse, and 7=very much worse. BL, baseline; Epti, eptinezumab; LS, least squares; MMRM, mixed model for repeated measures; MSQ, Migraine-Specific Quality of Life; PGIC, Patient Global Impression of Change; PI-MBS, patient-identified most bothersome symptom; SD, standard deviation; SE, standard error; VAS, visual analog scale; WPAI:M, Work Productivity and Activity Impairment, adapted for Migraine.

Supplemental Figure 1. Mean PI-MBS score (MMRM; efficacy analysis set)

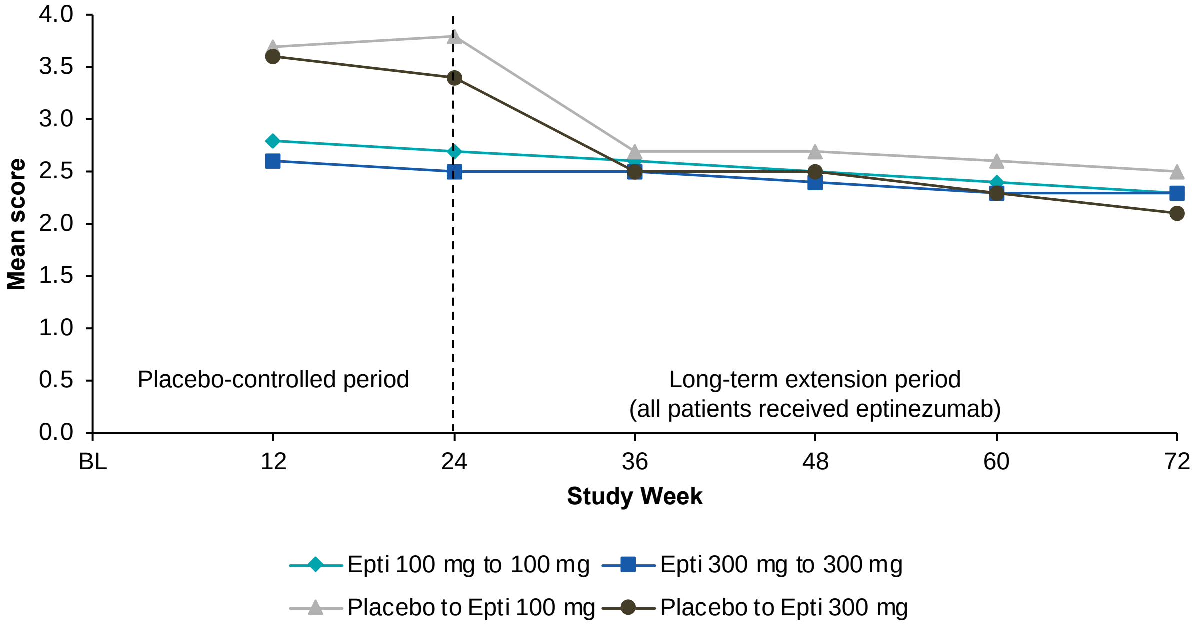

Epti, eptinezumab; MMRM, mixed model for repeated measures; PI-MBS, patient-identified most bothersome symptom.

Supplemental Figure 2. Mean PGIC score (MMRM; efficacy analysis set)

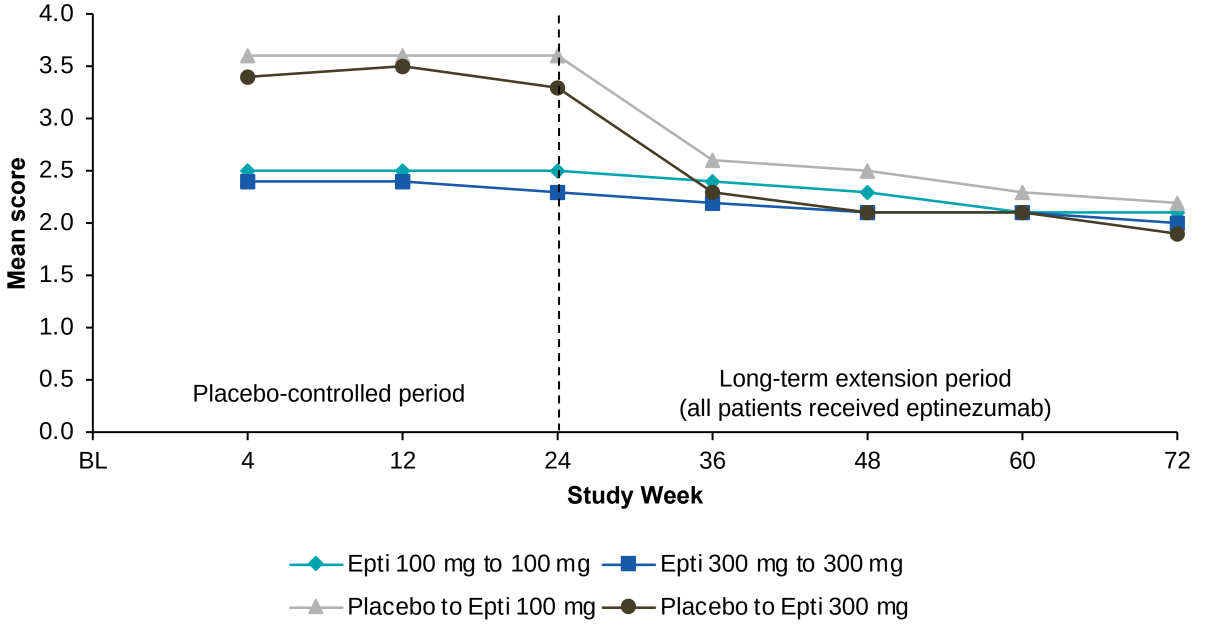

Epti, eptinezumab; MMRM, mixed model for repeated measures; PGIC, Patient Global Impression of Change.

Supplemental Figure 3. Mean change from baseline in EQ-5D-5L VAS score (MMRM; efficacy analysis set)

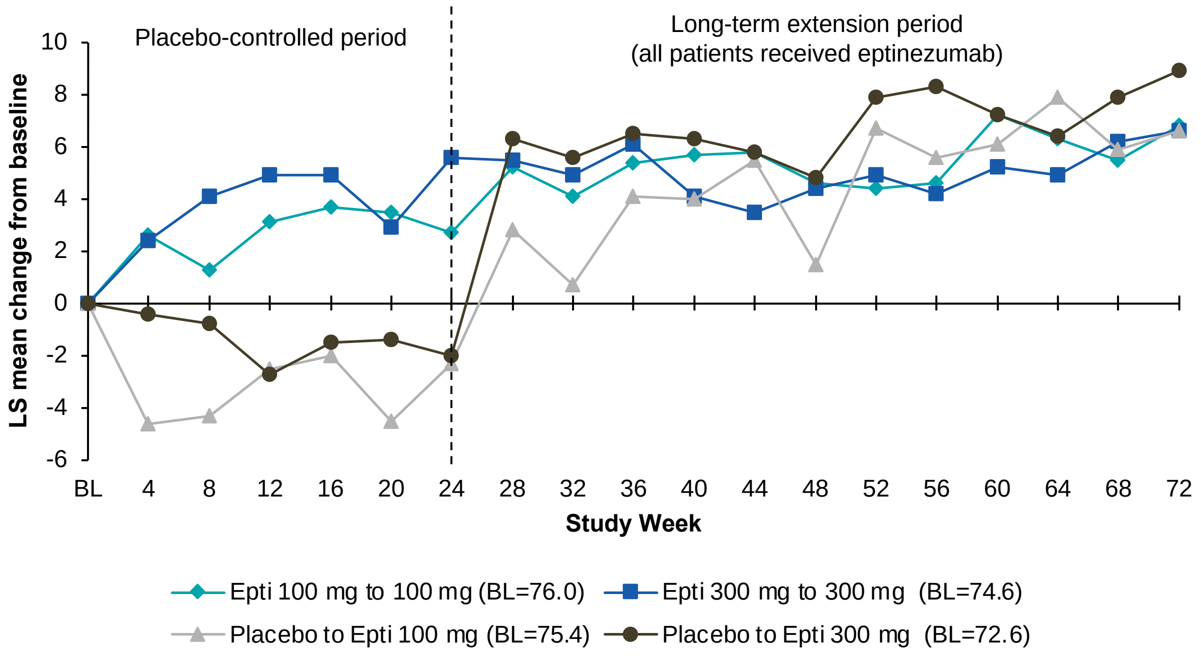

BL, baseline; Epti, eptinezumab; LS, least squares; MMRM, mixed model for repeated measures.
